# The Factor Structure of the CA-MIR as Evaluated Using Confirmatory Factor Analysis

**DOI:** 10.3389/fpsyg.2018.00190

**Published:** 2018-03-01

**Authors:** Paola Molina, Marta Casonato, Maria N. Sala, Silvia Testa

**Affiliations:** Department of Psychology, University of Turin, Turin, Italy

**Keywords:** CA-MIR, adult attachment, questionnaire, self-report, factor structure, confirmatory factor analysis

## Abstract

**Introduction:** The aim of the current study was to evaluate the factor structure of the CArtes- Modèles Individuels de Relations (CA-MIR), a self-report questionnaire designed to tap into the relational strategies of adults that was developed by a French-speaking research group coordinated by Blaise Pierrehumbert. The CA-MIR's particular merit lies in the richness and complexity of the theoretical model underpinning it. However, to date, this model has only been partially reproduced in studies using exploratory analysis and has never been tested via confirmatory factor analysis (CFA).

**Objective and Method:** We thus conducted CFA on data collected from a sample of 979 subjects, recruited using a snowball sampling method during the spring and fall of 2005. To assess if some item multidimensionality was present, we estimated both the independent clusters model (ICM-CFA) and a model in which some zero loading restrictions were removed.

**Results:** The results supported the originally proposed structure of the CA-MIR; the large majority of items were good indicators of the expected latent dimensions and only few items showed relevant secondary loadings or loaded in an unexpected factor. The instrument adequately differentiates the three attachment styles, taking into account both past and present experiences of attachment relationships, and providing a rich and complex assessment of multiple features of attachment. In terms of internal consistency, alpha values were satisfactory and comparable to those found in the original Swiss validation study.

**Conclusions:** Our results are of key importance for both research and clinical work, given the lack of valid and easy-to-administer tools for evaluating adult attachment.

## Introduction

Efforts to evaluate adult attachment have led to the production of a vast number of tools, both narrative and self-report. These instruments examine a diversity of contents (early attachment experience, current representations of attachment, romantic relationships, representations of caregiving) and are used in different fields of research: self-report tools have mainly been developed in social research contexts, and are generally administered to large samples (Crowell et al., 2008), while narrative instruments are more commonly adopted in developmental research, which are generally carried out on smaller samples and use meta-analysis to test their validity (van IJzendoorn, 1995; Hesse, 2008; Bakermans-Kranenburg and van IJzendoorn, 2009; Lucassen et al., 2011). Finally, different tools evaluate different levels of conscious awareness in relation to attachment representations: typically, narrative instruments such as the Adult Attachment Interview (AAI: George et al., 1984, 1985, 1996; Main and Goldwin, 1984; Main et al., 2002) facilitate evaluation of non-conscious aspects of attachment via the external coding of responses, while self-report questionnaires are centered on conscious ideas about attachment and relationships, and are generally not highly correlated with the AAI or other narrative instruments (Crowell et al., 1999, 2008).

The AAI is generally viewed as the benchmark tool for evaluations of adult attachment, although it is difficult and time consuming to administer, like all narrative instruments. Self-report tools are easier and more economical to use, but can be over-simplistic and therefore fail to access distinctive features of the different attachment styles, especially in relation to unconscious representations as in the case of dismissing attachment (Maier et al., 2004). The literature reflects general agreement on the fact that both types of measure are useful, each serving to evaluate different kinds of construct (Crowell et al., 2008): narrative instruments evaluate the “state of mind with respect to attachment,” based on infancy experience, while self-report instruments evaluate conscious representations of attachment in respect to different contents, that is to say, “attachment style” emerging from representations of early experience (Bartholomew and Horowitz, 1991). Despite their limitations, self-report instruments remain a valuable resource for clinical practice and research, particularly when large samples are involved.

The aim of our study is to evaluate the factorial structure of the CA-MIR (CArtes-Modèles Individuels de Relations: Pierrehumbert et al., 1996), a self-report instrument designed to tap into the attachment styles of adult, that can be used both as a traditional self-report questionnaire (Likert format), and as a Q-Sort instrument. In both versions, items are written on a card, and respondents are asked to put the cards in five piles, ordered from the less pertinent (“not at all true for me”) to the more pertinent (“very true for me”). In the Q-Sort version items are to be placed according to a forced distribution, resembling a normal distribution, while in the Likert format, the distribution is free.

The CA-MIR is a tool that shares aspects of both the above-mention research lines. In fact, one of its strengths lies in the opportunity it gives to be administered in a Q-Sort version that can solve at least some social desirability problems and be more effective in capturing even unaware aspects of individual representations. Nevertheless, in this study, only the Likert format is considered, since responses need to be independent in order to apply factor analysis tools.

Informed by attachment theory, the CA-MIR assumes that a self-and-other model drives personal relationships throughout life and adulthood: this model is proposed to have been generated by early experience with caregivers, although it may be modified by subsequent relational experiences. The questionnaire is intended for use with people of different ages (from adolescence to old age), and of both genders, regardless of family background and current family circumstances: parents or currently childless; currently or no longer living with family of origin; single or with a partner; coming from families with two parents, a single parent, or an adoptive family; with or without siblings.

The CA-MIR comprises 72 items designed to explore three different areas of representation: the present (questions about respondents' relationships with their current family unit), the past (questions concerning aspects of respondents' past experience with both parents, or with one parent in particular) and their *state of mind* (questions on respondents' current beliefs about the attachment relationship they had with their parents while growing up, as well as their semantic representations of parenting and the emotional needs of children and adults in general—the state of mind referred to here is that of which the respondent is consciously aware).

Representation of *Past* experience and *State of Mind* are two different concepts, because the first concerns the conscious representations of the past experience (namely the memories, the explicit narrative), the second the representations (even unconscious) about attachment, which derive but do not coincide with the past experience, as evidenced by the presence of *earned secure* adults (Hesse, 2008). *Present* representations, on the contrary, refers to the present experience, and are characterized by the actual experience with significant persons in the life of the subject (partners, children, parents). For a summary outline of the questionnaire's theoretical structure and some example of items, see Table 1.

**Table 1:** Theoretical structure of the instrument and sample items.

		**Past**		**Present**		**“State of Mind”**
**Preoccupied attachment** (E)	**A**	**Parental intrusion**	**B**	**Preoccupation with family**	**C**	**Resentment about childhood experience**
		As a child I was afraid of being abandoned. (39)		When I go away from those who are close to me, I feel ill at ease. (56)		I feel that I did not have the opportunity to assert myself in the environment I grew up in. (52)
**Autonomous attachment** (F)	**D**	**Parental support**	**E**	**Family support**	**F**	**Gratitude for support**
		Even if it is not true, I feel that I had the best parents in the world. (53)		I trust those who are close to me. (36)		I enjoy thinking about my childhood. (25)
**Dismissing attachment** (DS)	**G**	**Parental unavailability**	**H**	**Family distance**	**I**	**Resentment of childhood rejection**
		When I was a child, my wishes did not count much for the adults around me. (29)		I hate feeling dependent on others. (12)		Every time I try to think of my parents' good sides, I end up recalling their bad sides. (60)
**Unresolved attachment** (U)	**J**	**Parent-related trauma**			**K**	**Blocked memories**
		As a child, I was afraid of my parents. (33)				I have difficulty accurately recalling childhood events. (51)
**Structuration**	**L**	**Parental abdication**			**M**	**Regard for authority**
		When I was a child, I would set the grownups against one another to get what I wanted. (44)				In a family, it is very important that there be respect for the parents. (8)

For each of the three areas of representation, there are items characterizing three different attachment styles, namely *autonomous, preoccupied*, and *dismissing*, yielding a total of nine sub-scales. In addition, the groups of items concerning respondents' past experience of family relationships and their current *state of mind* both include two further sets of questions, related to *unresolved* attachment and respondents' *beliefs* about family relationships, respectively. This yields a further four sub-scales: two scales involve the presence of bereavement or traumas *unresolved*, an additional category that is attributed secondarily to the three main categories, as for the AAI. The two scales on *structuration* concern the ideas that people have regarding the education of the children and the authority: the educational principles experienced in the past (in the family of origin) and their own beliefs about education and authority. Although these last two scales are not envisaged as directly linked to attachment profiles, they offer useful additional information that can help to build up clinical profiles, e.g., when evaluating parenting skills.

In previous studies that adopted the Q-Sort format, the instrument showed good convergent and discriminant validity^1^; however, the factor structure was only partially reproduced in the Chilean validation study carried out by Garrido et al. (2009). Garrido and colleagues conducted a principal components analysis, identifying eight components: two of these exactly replicated the original scales labeled *Preoccupation with Family* (Preoccupied-Present) and *Family Support* (Autonomous-Present), while three factors partially replicated the original scales named: *Parental Abdication* (Structuration-Past), *Blocked Memories* (Unresolved-“State of mind”) and *Parental Intrusion* (Preoccupied -Past).

Considering the complexity of the CA-MIR theoretical model and the only partially overlapping results of the analyses just reviewed, additional analysis of the Likert-format items is necessary. The aim of the current study was to contribute to clarifying the factorial structure of the instrument, by performing confirmatory factor analysis (CFA) to directly verify the goodness-of-fit of the original 13-factor structure and assessing the concurrent and discriminant validity of the CA-MIR latent dimensions. According to the attachment literature and the item content, the following expectations were formulated about the latent dimensions' correlation. Due to a certain persistence of relational modalities across the life span, a moderate positive correlation between *Past, Present*, and *State of Mind* dimensions of Autonomous, Preoccupied, and Dismissing styles was expected; at the same time, these relations should have been stronger for *Past* and *State of Mind* dimensions because in both cases items deal with respondents' childhood experience. Autonomous style dimensions were expected to be negatively correlated with the dimensions of preoccupied and dismissing attachment styles, with weaker correlations when involving *Present* Autonomous style dimension because of the so-called *earned-secure* attachment patterns (Hesse, 2008). Moreover, Dismissing and Preoccupied dimensions were attended to be unrelated and *Blocked Memories* scale was expected to correlate with none of the Past dimensions since it deals with the lack in memories. Finally, we expected to find a moderate negative correlation between Autonomous style dimensions and the Past Structuration dimension related to the absence of authoritative parenting when respondent was a child: as highlighted by literature, “sensitive discipline” (van Zeijl et al., 2006) is a key aspect of secure experience in childhood.

## Methods

### Participants

We recruited 979 participants during the spring and fall of 2005, using a snowball sampling method. About one third of the sample (30.1%) was made up of people related to each other, such as siblings, partners, parents, and children. To reduce the potentially distorting effect of family ties among participants, we removed one member from each interrelated pair of respondents, two members from each interrelated group of three or four and three members from each interrelated group of five^2^. The percentage of interrelated respondents decreased to 0.9%, while sample size was reduced to 827 individuals (see Tables 2.1, 2.2).

**Table 2.1:** Sample (*n* = 827) compared to the Italian Population.

	**Sample *percentage***	**Italian popolation *percentage***
**Age**		ISTAT, 2006c
15-19	3.5	5.7
20-29	47.0	13.8
30-39	11.9	18.7
40-49	20.3	17.4
50-59	14.9	15.1
60 and over	2.4	29.3
**Gender**		ISTAT, 2006c
Percentage of females	61.2	51.9
**Highest level of education completed**		ISTAT, 2006a
Primary and middle school	23.1	64.3
High school	59.6	26.5
University	17.3	9.1
**Occupational status**		ISTAT, 2006a
Self-employed	13.2	11.9
In third party employment	45.8	32.7
Housewife	7.1	17.7
Student	29.3	8.9
Retired	3.1	21.9
Other (seeking employment and other categories of unemployed)	1.5	7.0
**Marital status**		ISTAT, 2006b
Single	51.0	43.9
Cohabiting	5.6	
Married	36.8	52.4
Divorced	5.2	1.2
Widowed	1.5	2.5
**Parenthood status** ***(percentage, n 405 selected to compare with Italian population)***		ISTAT, 2004
Having one or more children:	79.0	74.3
Number of children:		
1	43.4	47.8
2	46.3	41.3
3 or more	10.3	10.9

**Table 2.2:** Other sample characteristics (comparison data for the Italian population not available).

	**Sample percentage**
**Age** (min-max, M, *SD*)	15-81; 34.2; 13.4
**Birthplace**	
Northwestern Italy	80.2
Other	19.8
**Parenthood**	
Yes	40.4
No	59.6
**Parents alive**	
Mother	84.2
Father	72.1
**Issues faced during development**	
Early bereavement	1.0
Parental separation/divorce	1.3
Social disadvantage	0.2
Childhood adoption	0.1
Child of single parent	0.2
Parent-child separation	0.2
**Total**	**3.1**
**Recent issues**	
Illness or death of a loved one	1.7
Illness	0.1
Trauma	0.4
Separation/divorce	0.1
Psychosocial issues	0.4
**Total**	**2.7**

Most of the respondents were from Northwestern Italy (80.2%). Their ages ranged from 15 to 81 years (M = 34 years; *SD* = 13 years). Table 2.1 reports the sample's demographic characteristics, comparing them to those of the general Italian population: the 20–29 years age group was over-represented in the current sample (47.0%, as opposed to 13.8% of the overall Italian population) while the over 60s segment of the population was under-represented (2.4% in the current sample vs. 29.3% of all Italians; ISTAT, 2006c). The percentage of women was 61.2% (somewhat higher than in the Italian population: 51.9%). Compared to the general population (ISTAT, 2006a), the sample was better educated (more subjects with high school diplomas and university degrees), and contained a higher percentage of students and workers in third party employment; vice versa, those who had not completed their secondary education, as well as the categories of housewife, retired worker, job-seeker, and others outside of the active work-force were under-represented.

Fifty-one percent of the sample were single (vs. the lower level of 43.9% in the Italian population); 5.6% cohabited with a partner, 36.8% were married (vs. the higher level of 52.4% in the general population); the percentage of divorcees was higher than in the Italian population while the percentage of widowed persons was lower (ISTAT, 2006b). If we consider the family unit (*n* = 405)^3^, levels of parenthood and number of children were comparable to the general population (ISTAT, 2004). Table 2.2 reports the percentage of participants whose parents were still alive and the percentages of those reporting various types of recent and childhood challenges: in relation to these aspects, there was no general population data available for comparison.

With a view to test and cross-validate the factor structure of CA-MIR, we randomly divided the sample into two sub-samples^4^: Sample 1 (*N* = 419) and Sample 2 (*N* = 408).

### Procedure

The present study involved human beings in a data collection process aimed at gaining information about attitudes related to close interpersonal relationships and socio-demographical data from people aged 15 years old or more. The research conformed to the Codice Etico AIP (ethical code for research in psychology, Italian Psychological Association) and the provisions of the Italian laws on privacy and data protection (L. 196/2003). The participants volunteered to participate in the research, and signed an informed consent form in which they agreed to anonymously complete a Q-sort questionnaire and allow the researchers to use the data for scientific purposes. In case of minors, also parents' consent was collected.

The questionnaires were administered by undergraduate students who had received 20 h of *ad hoc* training. Each student administered five questionnaires in both Likert and forced Q-Sort formats, after obtaining participants' informed consent. The administration and coding procedure adopted were those recommended by the authors of the original instrument (Pierrehumbert, 2004). In the present study, only the Likert format data were analyzed.

### Instruments

#### CA-MIR questionnaire

The first author translated the questionnaire from French to Italian, discussing and revising the translation with the Swiss authors and with a colleague native speaker both in Italian and French, and producing Italian versions of the instrument for both male and female respondents^5^. The 72 items were scored on a 5-point Likert scale, from “not at all true for me” to “very true for me.” The Cronbach's Alpha for each of the 13 theoretical scale scores were the following. *Parental intrusion* (Preoccupied-Past, 6 items):.59; Preoccupation with family (Preoccupied-Present, 6 items):.70; Resentment about childhood experiences (Preoccupied-State of Mind, 6 items):.78; Parental support (Autonomous-Past, 6 items):.80; Family support (Autonomous-Present, 6 items):.72; Gratitude for support (Autonomous-State of Mind, 6 items):.75; Parental unavailability (Dismissing-Past, 6 items):.79; Family distance (Dismissing -Present, 3 items):.37; Resentment of childhood rejection (Dismissing -State of Mind, 6 items):.78; Parent-related trauma (Unresolved-Past, 6 items):.75; Blocked memories (Unresolved-State of Mind, 3 items):.67; Parental abdication (Structuration-Past, 6 items):.65; Regard for authority (Structuration-State of Mind, 6 items):.59.

#### Socio-demographic information

Respondents' socio-demographic data was collected concurrently, and each participant's family ties with other respondents, such as siblings, partners, parents, or children, were recorded. In addition, we collected information regarding non-normative life experiences that could potentially influence participants' attachment representations (e.g., juvenile grief, illness, divorce).

### Data analysis

On the data of Sample 1, the original 13-factor structure of CA-MIR was tested by a confirmatory factor model in which all cross-loadings were constrained to be zero, i.e., by means of an independent clusters model (ICM-CFA, Marsh et al., 2009). As reported in literature, ICM-CFA models are very restrictive and can produce biased estimates of factor correlations (Asparouhov and Muthén, 2009; Marsh et al., 2009, 2011, 2014; Morin et al., 2016). Non-target loadings motivated by substantive theory or by item formulation are typically present in multidimensional instruments and forcing them to zero can lead to overestimated factor correlations. This is particularly true when instruments assessing conceptually related constructs are involved (Morin et al., 2016). As concern the CA-MIR, several scales assess related constructs and we expected that some items exhibit “construct relevant psychometric multidimensionality” as defined by Morin et al. (2016). Some items of the Autonomous style scales could be weak reverse indicators of non-autonomous styles and the opposite, or some indicators of “Past” scales could be weak indicators of the “State of mind” scales and the opposite. Constraining these secondary loadings to be zero artificially increases the correlation between the corresponding factors. According to literature, the ICM-CFA solution need to be compared with the corresponding explorative factor model, following the exploratory structural equation modeling approach (ESEM, Asparouhov and Muthén, 2009; Marsh et al., 2014) to check for the presence of overestimated factor correlation.

In the present work, it was not possible to apply the ESEM approach because the high number of latent factors (13) would have required the estimation of more parameters than the number of participants (930 free parameters); and neither it was feasible to identify the subset of items affected by multidimensionality on theoretical grounds. We thus performed an exploratory factor analysis with target rotation and Minres as a method of estimation, followed by a CFA in which all the EFA secondary loadings >0.30, alongside the target loadings, were estimated (called complex-CFA model in the following)^6^. To identify the latent variables scale, factor variances were set to 1. Given that the data violated the multinormality condition^7^, we used the Maximum Likelihood method (ML) to estimate the parameters, correcting the chi-square and standard errors (Satorra and Bentler, 1994). The following cut-off criteria were used to establish the model's goodness of fit: RMSEA < 0.08; CFI > 0.95; SRMR < 0.08 (Browne and Cudeck, 1993; Hu and Bentler, 1995, 1999); the Satorra-Bentler scaled difference χ^2^ test (SBDiff, Satorra and Bentler, 2001) was used to compare the fit of nested CFA models.

In the second sample, we cross-validated the model emerged from the analyses of the data from the first sample.

All analyses were conducted using SPSS 24; R 3.4; Prelis and LISREL 8.72 (Jöreskog and Sörbom, 1996a,b).

## Results

### Factor analyses on sample 1

The theoretical 13 factor model showed good overall fit to the data as all the fit indices, RMSEA, CFI, and SRMR, fulfiled the cut-off values (Table 3). The loadings were all statistically significant (*p* < 0.01) and only five of them were below 0.30. Several factors were highly correlated, in particular correlation was > 0.90 (in absolute value) for scales A and C, C and I, D and F, F and I, G and I and 7 correlation values were in the range 0.80–0.90.

**Table 3:** Goodness of fit of the CFA models on Sample 1.

**Model**	**SB Chi-square**	***DF***	**SBDiff**	***DF***	***p***	**RMSEA (CI)**	**CFI**	**SRMR**
13 factor ICM-CFA	4186.8	2406				0.042 (0.040–0.044)	0.95	0.067
13 factor Complex-CFA model	3635.8	2376	389.9	30	<0.0001	0.036 (0.033–0.038)	0.96	0.063
12 factor Complex-CFA model	3694.9	2392	54.5	16	<0.0001	0.036 (0.034–0.038)	0.96	0.064

Based on the results of the explorative target rotated factor analysis reported in Appendix, a second CFA model that included secondary loadings >0.30 (in absolute value) was performed. As depicted in Table 3, this second model outperformed the previous one, and the Satorra Bentler scaled difference χ^2^ test (SBDiff) was statistically significant. Moreover, the number of factor correlations in absolute value above 0.80 decreased from 12 to 4, with maximum values for scales D and F (0.88), and scales G and I (0.86).

In the light of these results, a third model with 12 latent variables in which scales D and F belongs to the same latent factor was estimated, in order to assess whether the two highest correlated latent factors could be collapsed. As shown in Table 3, the worsening of model fit passing from 13 to 12 latent factors was statistically significant, suggesting retaining the 13 complex-CFA model.

### Cross-validation on sample 2

In order to gain further understanding of, and further clarify, the factor structure of the CA-MIR, we replicated the complex-CFA model in the second sample.

The overall fit statistics were good: SB-Chi-Square (2376) = 4130.6, *p* < 0.001, RMSEA = 0.043; 90% CI = 0.040–0.045, CFI = 0.96, SRMR = 0.067. As summarized in Table 4, the large majority of the items loaded on the target factor with values >0.30.

**Table 4:** Complex-CFA model: Standardized parameter estimates (Sample 2, *N* = 408).

	**Preoccupied**			**Autonomous**			**Dismissing**			**Unresolved**		**Structuration**	
	**A**	**B**	**C**	**D**	**E**	**F**	**G**	**H**	**I**	**J**	**K**	**L**	**M**
7	**0.18**	-	**0.30**	–	–	–	–	–	–	–	–	–	–
35	**0.28**	–	**0.46**	–	–	–	–	–	–	–	–	–	–
39	**0.15**	–	–	–	–	**−0.39**	–	–	–	–	–	–	–
48	**0.36**	–	–	–	–	–	–	−0.07	–	–	–	–	–
54	**0.72**	–	–	0.03	–	–	–	–	–	–	–	–	–
62	**0.51**	–	–	–	–	–	–	–	–	–	–	–	–
2	–	–	**0.68**	–	–	–	–	–	–	–	–	–	–
26	–	–	**0.25**	–	–	–	–	–	**0.41**	–	–	–	–
41	–	–	**0.53**	–	–	–	–	–	–	–	–	–	–
52	–	–	**0.63**	–	–	–	–	–	–	–	–	–	–
55	–	–	**0.71**	–	–	–	–	–	–	–	–	–	–
64	**0.34**	–	**0.46**	–	–	–	–	–	–	–	–	–	–
20	–	**0.51**	–	–	–	–	–	–	–	–	–	–	–
22	–	**0.56**	–	–	–	–	–	–	–	–	—	–	–
32	–	**0.47**	–	–	–	–	**−0.21**	–	–	–	–	–	–
56	–	**0.56**	–	–	–	–	–	–	–	–	–	–	–
68	–	**0.59**	–	–	–	–	–	–	–	–	–	–	–
72	–	**0.56**	–	–	–	–	–	–	–	–	–	–	–
9	–	–	–	**0.64**	–	–	−0.14	–	–	–	–	–	–
21	–	–	–	**0.56**	–	0.11	–	–	–	–	–	–	–
40	–	–	**−0.30**	**0.37**	–	–	–	–	–	–	–	–	–
53	–	–	–	**0.32**	–	**0.25**	–	–	–	–	–	–	–
58	–	–	–	**0.55**	–	–	–	–	–	–	–	–	–
66	–	–	–	**0.46**	–	–	**−0.30**	–	–	–	–	–	–
10	–	–	–	**0.27**	–	0.14	–	–	–	–	–	–	–
11	–	–	–	–	–	**0.54**	–	–	–	**−0.37**	–	–	–
19	–	–	–	**0.38**	–	**0.37**	–	–	–	–	–	–	–
25	–	–	–	–	–	**0.65**	–	–	–	–	–	–	–
28	–	–	–	–	–	**0.51**	–	–	–	–	–	–	–
6	–	–	–	–	**0.61**	0.12	–	–	–	–	–	–	–
1	–	–	–	–	**0.53**	–	–	–	–	–	–	–	–
4	–	–	–	–	**0.55**	–	–	–	–	–	–	**0.24**	–
18	–	–	–	–	**0.14**	–	–	–	–	–	–	–	–
27	–	–	–	–	**0.63**	–	–	–	–	–	–	–	–
36	–	–	–	–	**0.73**	–	–	–	–	–	–	–	–
69	–	–	–	–	**0.65**	–	–	–	0.00	–	–	–	–
15	–	–	–	–	–	–	0.03	–	–	**0.73**	–	–	–
29	–	–	–	–	–	–	**0.75**	–	–	–	–	–	–
30	–	–	–	–	–	–	**0.63**	–	–	–	–	–	–
31	–	–	–	–	–	–	**0.27**	–	–	–	–	**0.41**	–
38	–	–	–	–	–	–	**0.52**	–	–	–	–	–	–
71	–	–	–	**−0.42**	–	–	**0.35**	–	–	–	–	–	–
13	–	–	–	–	–	–	–	–	**0.62**	–	–	–	–
47	–	–	–	–	–	–	–	–	**0.58**	–	–	–	–
50	–	–	–	–	–	–	–	–	**0.42**	–	–	–	–
57	–	–	0.18	–	–	–	–	–	**0.53**	–	–	–	–
60	–	–	–	–	–	–	–	–	**0.63**	–	–	**0.18**	–
67	–	–	–	–	–	–	−0.01	–	**0.71**	–	–	–	–
12	–	–	–	–	–	–	–	**0.79**	**−0.33**	–	–	–	–
14	–	–	–	–	–	–	–	**0.56**	–	–	–	–	–
17	–	–	–	–	–	–	–	**0.20**	–	–	–	–	–
3	–	–	–	–	–	–	–	–	–	**0.48**	–	–	–
33	–	–	–	–	–	–	–	–	–	**0.61**	–	–	–
45	–	–	–	–	–	–	–	–	–	**0.62**	–	–	–
59	–	–	–	–	–	–	–	–	–	**0.69**	–	–	–
61	–	–	–	–	–	–	–	–	**0.51**	−0.02	–	0.16	–
63	–	–	–	–	–	–	–	–	–	**0.61**	–	–	–
37	–	–	–	–	–	–	–	–	–	–	**0.66**	–	–
46	–	–	–	–	–	–	–	–	–	–	**0.48**	–	–
51	–	–	–	–	–	–	–	–	–	–	**0.71**	–	–
5	–	–	–	–	–	–	–	–	–	–	–	**0.57**	–
16	–	–	–	–	–	–	–	–	–	**0.29**	–	**0.53**	–
23	–	–	–	–	–	–	−0.05	–	–	–	–	**0.60**	–
42	–	–	–	–	–	–	–	–	–	–	–	**0.38**	–
44	–	–	–	–	–	–	–	–	–	–	–	**0.30**	–
70	–	–	–	–	–	–	–	–	–	–	–	**0.47**	–
8	–	–	–	–	–	–	–	–	–	–	–	–	**0.67**
24	–	–	–	–	–	–	–	–	–	–	–	–	**0.16**
34	–	–	–	–	–	–	–	–	–	–	–	–	**0.53**
43	–	–	–	–	–	–	–	–	–	–	–	–	**0.46**
49	–	–	–	–	–	–	–	–	–	–	–	–	**0.51**
65	–	–	–	–	–	–	–	–	–	–	–	–	**0.65**

*A, Parental intrusion; B, Preoccupation with family; C, Resentment about childhood experiences; D, Parental support; E, Family support; F, Gratitude for support; G, Parental unavailability; H, Family distance; I, Resentment of childhood rejection; J, Parent-related trauma K, Blocked memories; L, Parental abdication; M, Regard for authority*.

*Statistically significant (p < 0.05) factor loadings are highlighted in bold*.

Items 7, 35, 39 resulted to be poor indicators of factor A (Preoccupied-Past); their loadings, albeit statistically significant, were low in value. More specifically, item 7 (“I' d like my children to be more autonomous than I have been”) and item 35 (“My parents didn 't really realize that a child who is growing up needs to have a life of his own”) seemed to be indicators of the State of Mind dimension instead of the Past, whereas item 39 (“As a child, I was scared of being abandoned”) loaded heavily (and negatively) on Autonomous-State of Mind factor.

Items 26 (“As a teenager, no one around me has ever really understood my worries”) and 64 (“In my family, we lived isolated from the rest of the world”) loaded both on the expected factor (scale C), and on a second factor: factor A (item 26), and factor I (item 64).

As regard autonomy factors (D, E, and F), item 40 (“As a child, I was encouraged to share my feelings”) loaded also on scale C, but with negative sign, and item 53 (“Even if it's not true, I have the feeling l've had the best parents in the world”) showed a secondary positive loading on factor F. Moreover, item 18 (“I spend a lot of time talking to the people who are close to me”) was a poor indicator of factor E (Autonomous-Present), without showing relevant secondary loadings, and item 10 (“I believe l've known how to give back to my parents the love they've given me”) was more an indicator of the Past than of the State of Mind dimension.

Moving to the Dismissing dimensions (factors G, H, and I), item 15 (“When I was a child, the people close to me were often impatient and irritable”) resulted to be related to the J factor instead of the G factor and item 31 (“When I was a child, it was difficult for us to make decisions in the family”) to factor L. As regard item 12 (“I hate feeling I depend on others”), an unexpected negative secondary loading on factor I was observed.

In the remaining four scales (factors J, K, L, and M), only two items showed poor fit: item 61 (“I have the feeling I was a rejected child”), that resulted to be an indicator of factor I instead of factor J, and item 24 (“Adults have to control their emotions with their children, whether it concerns pleasure, love or anger”), that emerged as a weak indicator of scale M without showing any secondary loadings.

In summary, four items (17, 18, 24, and 44) were weak indicators of the intended factor and did not load on secondary factors. Others four items (10, 15, 61, and 6) did not load on the expected factor but on a plausible related factor; and finally, five items (64, 11, 19, 71, and 12) resulted in a substantial loading (>0.30) on two factors.

As reported in Table 5, six correlations were >0.80 in absolute value, and two of them were >0.90 (the positive one between factors D and F and the negative one involving scale I and D). The signs of the correlations were in line with the theoretical expectation: The Past and State of Mind dimensions of each attachment style were positively associated with one another; Autonomous Attachment was negatively associated with *Preoccupied* and *Dismissing* State of mind dimensions, while these last were positively associated with one another. Only for the Autonomous Attachment style there were a strong correlation with the Present dimension (0.69 and 0.70 for Past and State of Mind dimensions, respectively).

**Table 5:** Complex-CFA model: factors correlations (Sample 2, *N* = 408).

	**Preoccupied**			**Autonomous**			**Dismissing**			**Unresolved**		**Structuration**	
	**A**	**B**	**C**	**D**	**E**	**F**	**G**	**H**	**I**	**J**	**K**	**L**	**M**
A	1												
B	**0.20**	1											
C	**0.68**	**0.20**	1										
D	**−0.36**	0.12	**−0.65**	1									
E	**−0.37**	0.12	**−0.52**	**0.69**	1								
F	**−0.38**	0.09	**−0.56**	**0.90**	**0.70**	1							
G	**0.46**	0.20	**0.89**	**−0.79**	**−0.51**	**−0.63**	1						
H	**0.19**	−0.01	**0.44**	**−0.41**	**−0.43**	**−0.35**	**0.54**	1					
I	**0.56**	0.03	**0.78**	**−0.93**	**−0.73**	**−0.86**	**0.87**	**0.54**	1				
J	**0.59**	0.04	**0.66**	**−0.81**	**−0.55**	**−0.62**	**0.76**	**0.38**	**0.85**	1			
K	0.04	0.13	**0.27**	**−0.14**	**−0.14**	**−0.26**	**0.32**	0.10	**0.15**	−0.01	1		
L	0.15	0.14	**0.36**	**−0.42**	**−0.46**	**−0.63**	**0.53**	**0.27**	**0.51**	**0.38**	**0.23**	1	
M	**−0.22**	**0.25**	**−0.13**	**0.29**	**0.41**	**0.54**	**−0.18**	0.14	**−0.23**	**−0.23**	−0.10	**−0.36**	1

*A, Parental intrusion; B, Preoccupation with family; C, Resentment about childhood experiences; D, Parental support; E, Family support; F, Gratitude for support; G, Parental unavailability; H, Family distance; I, Resentment of childhood rejection; J, Parent–related trauma K, Blocked memories; L, Parental abdication; M, Regard for authority. Values statistically significant at p < 0.05 are in bold*.

With regard to Scales J, K, L, and M, the only correlations above 0.30 were that between J and L (0.38), and L and M (−0.36). Factors J and L were also strongly correlated with the attachment style scales (especially the Autonomous and Dismissing scales), whereas scales K and M were those least strongly correlated with all the other scales.

## Discussion

### Factorial structure of the CA-MIR

In the evaluation of the factorial structure of the CA-MIR, both the ICM-CFA model and a model with the presence of several secondary loadings were estimated. The former was aimed at directly assessing the goodness of fit with the Italian data of the 13-factor theoretical model; the latter, here called complex-CFA model, was considered to eventually take into account the presence of item multidimensionality (Marsh et al., 2014; Morin et al., 2016).

The comparison of the two above-mentioned models in the first sample showed that several secondary loadings were present. The same model, replicated in the second sample, confirmed the presence of some multidimensionality: in a few circumstances, secondary loadings were greater than the loadings in the expected factors and others few items showed not negligible cross-loadings. The content of these unfitting items justifies these findings: in most cases, the items ambiguity was connected to the distinction between Past and State of mind scales of the same attachment style or to a negative relation with a scale of a different attachment style (indicators of Autonomous style with negative loadings on one of the unsecure styles, and vice versa). Four items emerged as poor indicators of the expected latent dimension, without exhibiting secondary loadings. One of these, item 17, belongs to the *Family distance* scale and its misfit could be responsible for the very low internal consistency of the scale scores.

The internal consistency of each of the remaining scales was satisfactory and comparable to that found in the original Swiss validation study. Only *Family distance* scale displayed an unsatisfactory value (0.37), but this had also been the case for the Chilean sample (0.33, Garrido at al., 2009) and the Swiss sample (0.48, Pierrehumbert et al., 1996).

The CFA approach allowed us to directly test and confirm the 13-factor model and contributes to advancing our understanding of why exploratory tool like that applied in Garrido et al. (2009) could tend to portray the CA-MIR as measuring a lower number of dimensions. The extent of the inter-correlations among the 13 scales, the presence of items that load negatively on secondary factors and of some misplaced items could be the reason why in the Chilean study only eight dimensions were obtained.

### Convergent and discriminant validity of the 13 subscales

If we examine the correlations among the scales in light of the theoretical model, we find that the CA-MIR can differentiate between the three main styles of attachment. The signs of the correlation coefficients are theoretically coherent: specifically, the three scales associated with autonomous attachment are negatively correlated with those assessing preoccupied and dismissing attachment, which in turn are positively correlated with one another. The last-mentioned result invites further analysis: indeed, the relationship between the two insecure styles is puzzling and requires cautious interpretation.

Within each attachment style, the correlations among individual scales are positive, but vary in strength. *Past* and *State of Mind* factors are strongly correlated for each of the three attachment patterns. These robust associations could be due to the formulation of the *State of Mind* items, which frequently refer to the past. Indeed, as conceived by the authors of the CA-MIR, the *State of Mind* scales typically investigate respondents' current evaluations of their childhood experience *(52: “I feel that I did not have the opportunity to assert myself in the environment I grew up in”)* (Pierrehumbert et al., 1996).

The correlations between the dimension of the *Present* and the other two dimensions (*Past, State of Mind*) differ across the three attachment styles: these correlations are high (>0.60) for autonomous attachment patterns, moderate (around 0.50) for dismissing attachment and low (0.20) for preoccupied attachment. These differences in the correlation of the three dimensions (*Past, Present*, and *State of Mind*) may be interpreted in light of item semantics. Nevertheless, a theoretical explanation is also plausible: the *Past* and *State of Mind* scales concern child experiences, whereas the *Present* scales regard relationships within the respondent's current family unit, often involving a partner and a different type of relationship (romantic). However, the stronger association among *Present, Past*, and *State of Mind* dimensions for the autonomous attachment style could imply that the CA-MIR is not effective in identifying so-called *earned-secure* attachment patterns. Individuals with earned-secure attachment describe negative childhood experiences, which might be expected to produce some form of insecure attachment, but are nonetheless found to display a secure/autonomous state of mind (Hesse, 2008).

The last four Scales concern two further constructs.

Scales J and K are theoretically related to traumatic experience and are designed to tap into *Unresolved* or *Disorganized* attachment. Scales J and K were not correlated with one other: the former concerns extremely negative childhood experiences (the mean score obtained by our sample on this scale was very low), while the latter refers to gaps in memory and consequently does not appear to be linked to any of other scales, particularly those concerning the past. In contrast, Scale J was positively correlated with Scale L (*Parental Abdication*), providing support for the notion that negative experience in childhood may also be related to a lack of sensitive discipline. Although this correlation is interesting, it is difficult to view Scales J and L as two facets of the same unresolved attachment style: they must be interpreted in terms of individual profiles.

Scales L and M assess respondents' general views about the role of parents and the importance of authority: theoretically, these scales are not linked to attachment but to “ideological”/“abstract” assumptions about parents and adults. In the current dataset, these scales clearly loaded onto distinct factors, and the pattern of correlations confirms their independent status.

Scales L and M were moderately and negatively correlated, as expected: the former concerns the absence of parental discipline in respondents' childhood experience, and the latter, on the contrary, their views regarding the importance of authority.

Scales J (*Parent-related trauma*) and L (*Parental abdication*) shared the same correlation profile: they were positively correlated with both types of insecure attachment and negatively correlated with autonomous attachment. This finding suggests that Scale J reflects parental behavior that is highly inadequate rather than just traumatic: according to the attachment literature, markedly negative childhood experience may be related to insecure attachment.

The Scale L correlations are lower, but in the same direction: this provides support for the idea that “sensitive discipline” (Van Zeijl, et al., 2006) is a key aspect of secure experience in childhood.

Scale M (*Regard for Authority*) displayed an inverse pattern of associations, but the correlations were markedly weaker. Thus, the CA-MIR appears to be able to distinguish attachment security from ideological beliefs concerning the family.

## Conclusion

This study is part of a broader research project aimed at completing Italian standardization of the CA-MIR. The objective of the current study was to evaluate the factor structure of the instrument. Indeed, the CA-MIR is an interesting tool precisely because of the richness and complexity of its theoretical model. Nevertheless, this model had never been tested with CFA, whereas previous exploratory analysis had only partially reproduced the original theoretical model (Garrido et al., 2009).

Our CFA enabled us to confirm the originally proposed structure of the CA-MIR: when used with its Likert response format it adequately differentiates between the three attachment styles, taking into account both past and present experiences of attachment relationships, and providing a complex and rich assessment of multiple features of attachment.

From a cross-cultural point of view, our results support the original theoretical model in a novel cultural context, namely Italy. The importance of this generalization is linked to one of the core hypotheses of the attachment theory: the broad cross-cultural stability of attachment representations. Therefore, the 13 scores obtained by administering the CA-MIR may be taken as valid indexes providing key information about subjective experience and conscious representations of attachment.

Taken as a whole, the Italian internal consistency values are acceptable and comparable to those obtained in the original Swiss validation study. As mentioned earlier, Scale H displays a number of weakness, including both the low number of items composing the scale (three items vs. the six items forming most of the other scales) and the formulation of Item 17. The last-mentioned item (“*It's better not to lament a bereavement too much so as to get over it faster*”), could be replaced by another question that more unambiguously taps into currently deployed dismissing attachment strategies.

The outcomes of the correlation analysis provide some pointers regarding appropriate interpretation of CA-MIR scores. Firstly, we must be cautious in differentiating between respondents' evaluations of past experience and their states of mind about attachment: these two dimensions are strongly correlated for all attachment styles, and individual cases of difference between these scores must be carefully assessed. The dimension of present experience is relatively independent of past experience and state of mind, particularly in relation to dismissing and preoccupied attachment styles, and this is in line with the theoretical expectations. Furthermore, due to the strong correlation between dismissing and preoccupied attachment scales, we must be cautious in viewing them as distinct.

Scales J and K are not correlated and must therefore be taken as two separate indicators rather than as forming a single scale of unresolved attachment, but they can nonetheless be of assistance in interpreting a respondent's clinical profile. Although the L and M scales are not envisaged as directly linked to attachment profiles, they offer useful additional information that can help to build up clinical profiles, e.g., when evaluating parenting skills.

These characteristics, combined with the advantages of the self-report format, make the CA-MIR a useful and powerful instrument, capable of discriminating between representations of secure and insecure attachment, and of providing information about attachment representations that is of both clinical and research value.

The sampling procedure adopted in the current study is one of its limitations. We chose a snowball sampling method in order to recruit a large sample. However, starting out from a northern region and a young adult population, this procedure reduced the representativeness of the sample because of the high proportion of participants from North-western Italy and aged between 20 and 29 years. Nevertheless, we have provided a detailed comparison of the demographic characteristics of our sample with those of the general Italian population, highlighting both differences and similarities, to facilitate contextualization of our findings. Although not completely representative, our sample is comparable to that of the original Swiss-led validation study, and its principal limitation (the over-representation of young adults) may also be viewed as an interesting feature, given that individuals in this age range typically face the key attachment challenges of adult life such as developing romantic relationships, growing more independent of their parents and becoming parents themselves. More in-depth analysis of socio-cultural variables is one of our future research objectives. Moreover, sample size was not big enough to employ the ESEM approach. The comparison between the ICM-CFA and complex-CFA models showed that some “construct relevant item multidimensionality” (Morin et al., 2016) affects the structure of the CA-MIR leading to inflated factors correlations if ignored and it could not be excluded that some item multidimensionality remained unmodeled in the present study. However, results confirm the distinctiveness of the 13 factors, potentially overestimating their correlation and the comparison with the ESEM model is a further research goal to carry out when a bigger sample will be available.

In addition, an interesting direction for future research could be to test the concurrent and discriminant validity of the Italian version of the test, by comparing the CA-MIR with other instruments assessing adult attachment. In particular, it would be useful to compare CA-MIR self-report data with outcomes obtained using narrative instruments, such as the AAI. Comparison of AAI categories with CA-MIR scores would be a further test of the CA-MIR's effectiveness in investigating attachment representations, and can be useful to further analyse the behavior of the few unfitting items observed in this study. Finally, the instrument could be administered to clinical groups in order to assess its ability to detect specific aspects of insecure attachment in particular sub-samples, as has already been done in previous studies (Molina et al., 2007, 2009) with a small group of maltreating parents.

## Author contributions

PM: Conceived the study and wrote the manuscript; MC and MNS: Contributed to data collection, to analyze the data and to revise the manuscript; ST: Analyzed the data and contributed to revise the manuscript.

### Conflict of interest statement

The authors declare that the research was conducted in the absence of any commercial or financial relationships that could be construed as a potential conflict of interest.

## Footnotes

^1^In terms of convergent validity, Roskam et al. (2011) found a relationship between secure attachment as evaluated using the CA-MIR in both mothers and fathers and secure attachment in children referred for externalizing behavior problems at 4 years, as assessed using the French version of Attachment Q-Sort (Pierrehumbert et al., 1995a,b). The instrument also showed discriminant validity with respect to measures of a range of psychopathological constructs in studies with clinical samples: psychotic patients (Benony et al., 2001), adolescents with eating disorders and substance abuse (Pierrehumbert et al., 2002; Miljkovitch et al., 2005), and groups with a range of different disorders (Lacasa et al., 2015).

^2^Given that we had not followed any particular criterion for order of data entry, we removed from the data set the first subject entered for each interrelated pair, the first and third subjects entered for interrelated groups of three or four and the first, third and fifth subjects entered for groups of five.

^3^To enable comparison with the general population, we followed the ISTAT (National Institute of Statistics) definition of family unit, taking into account participants who were married, cohabiting, divorced or widowed.

^4^We checked the equivalence of the two groups, finding no significant differences in relation to the following variables: age, gender, highest level of education completed, birthplace, marital status, parenthood status, issues encountered during development, issues encountered recently and semester in which the instrument was completed (all questionnaires were administered during the same calendar year, 2005, but across two different academic years: 2004/2005—II semester, and 2005/2006—I semester).

^5^The original French version and the Italian and English versions of the questionnaire are available at https://sites.google.com/site/bpierreh/home/instruments/camir.

^6^We tested models with less liberal thresholds (0.29, 0.25) but they resulted in some items with suspiciously high standard error of estimates compared to the others.

^7^In presence of ordinal data, polychoric correlations and WLS or DWLS estimators are usually suggested. As a control analysis we performed a DWLS estimation based on polychoric correlations on Sample 1 obtaining comparable values for global fit indices but some Heywood cases emerged, whereas MLR resulted in proper solutions; for this reason we considered the MLR results as more reliable.
