# Supplementary material for: The Factor Structure of the CA-MIR as Evaluated Using Confirmatory Factor Analysis
Source: Front Psychol. 2018 Mar 1;9:190. doi: 10.3389/fpsyg.2018.00190 (PMC5839230; doi:10.3389/fpsyg.2018.00190)
Supplement: Supplementary file 1 [file Appendix.pdf]

Appendix – Loadings of the explorative factor analysis with target rotation (target loadings and cross-loadings above .30 are in bold)

	Preoccupied			Autonomous			Dismissing			Unresolved		Structuration	
Item	A	B	C	D	E	F	G	H	I	J	K	L	M
7	**0.21**	0.02	**0.44**	-0.03	0.07	0.04	0.06	0.02	0.20	-0.09	0.09	-0.12	0.06
35	**0.47**	0.06	**0.46**	-0.07	0.13	0.04	0.09	0.15	0.27	0.26	0.02	-0.03	-0.19
39	**0.35**	0.05	0.08	0.19	0.03	**-0.31**	-0.01	-0.06	0.17	0.05	0.06	0.01	0.14
48	**0.40**	0.16	0.02	0.02	0.12	-0.27	0.09	**-0.31**	-0.07	-0.08	0.12	0.20	0.09
54	**0.77**	-0.10	0.28	**0.44**	-0.09	0.13	0.00	0.05	0.19	0.08	0.15	0.19	0.01
62	**0.59**	-0.17	0.18	0.08	-0.08	0.30	0.05	-0.06	0.00	0.06	0.08	0.06	0.08
2	0.30	0.00	**0.56**	-0.13	0.18	0.14	0.04	0.01	0.17	-0.08	0.01	-0.21	-0.14
26	0.23	-0.13	**0.29**	0.27	0.04	0.16	0.17	0.00	**0.44**	0.05	0.07	0.01	0.12
41	0.22	0.11	**0.43**	-0.02	0.07	0.12	0.04	0.04	0.18	0.11	0.10	0.03	-0.03
52	0.02	0.06	**0.19**	0.04	0.13	0.08	0.14	0.11	0.15	0.00	0.13	-0.04	-0.07
55	0.24	0.01	**0.37**	0.11	0.04	0.15	0.25	0.01	0.05	0.11	-0.02	-0.06	0.03
64	**0.34**	0.04	**0.43**	0.11	-0.12	0.19	0.12	-0.04	0.07	0.24	-0.05	0.06	0.12
20	-0.17	**0.65**	0.09	0.24	0.30	0.09	0.01	0.08	0.11	0.09	0.19	0.00	0.06
22	0.01	**0.72**	-0.02	0.20	0.01	0.07	0.09	0.08	0.10	-0.16	0.07	0.08	0.21
32	0.06	**0.71**	-0.13	0.21	0.05	0.15	**-0.36**	0.02	0.17	-0.04	0.00	0.19	-0.03
56	0.06	**0.75**	0.03	0.15	0.09	-0.03	0.06	-0.01	0.15	0.06	0.00	0.09	0.18
68	0.04	**0.87**	-0.07	0.17	0.23	0.07	-0.06	-0.11	0.01	-0.14	0.14	0.00	0.16
72	0.02	**0.71**	-0.06	0.18	0.09	-0.17	-0.06	-0.06	0.14	-0.10	-0.08	0.06	0.16
9	0.03	0.09	0.08	**0.49**	0.08	0.19	**-0.32**	-0.05	-0.15	-0.07	0.03	0.02	0.09
21	0.12	0.11	0.02	**0.66**	0.05	**0.36**	-0.22	0.15	-0.11	0.09	0.06	0.10	0.14
40	0.08	0.03	**-0.31**	**0.37**	0.27	0.29	-0.23	-0.08	-0.05	0.16	0.07	0.17	0.06
53	0.15	0.21	0.07	**0.47**	0.14	**0.35**	0.06	0.03	-0.09	-0.19	0.02	0.04	0.04
58	-0.27	0.02	-0.18	**0.56**	0.12	0.12	0.10	0.09	-0.15	0.07	0.02	0.09	-0.05
66	0.23	-0.05	-0.03	**0.63**	-0.14	0.21	**-0.43**	0.02	0.01	-0.08	0.08	0.02	-0.03
10	-0.07	0.08	0.01	**0.35**	0.04	**0.39**	0.30	0.03	0.16	0.05	0.04	0.01	0.06
11	0.02	0.02	0.02	0.15	0.08	**0.25**	-0.26	0.02	-0.17	**-0.32**	0.07	0.04	0.09
19	0.27	0.10	0.08	**0.41**	0.25	**0.22**	-0.11	0.07	-0.17	0.00	0.03	0.05	0.04
25	-0.22	0.17	0.03	0.20	0.08	**0.36**	-0.19	-0.05	0.07	-0.04	0.23	0.07	0.15
28	-0.03	0.04	-0.13	0.20	0.12	**0.43**	0.07	0.11	0.02	0.04	0.04	0.07	0.21
6	0.00	0.02	0.16	0.16	**0.50**	**0.16**	0.05	0.20	0.06	0.17	0.10	0.11	0.16
1	-0.01	0.06	0.02	-0.11	**0.81**	0.25	0.02	-0.19	0.05	0.01	0.01	0.18	0.02
4	0.14	0.04	0.04	0.00	**0.71**	0.19	-0.01	0.18	0.12	0.03	0.05	**0.35**	0.08
18	0.12	0.01	-0.06	-0.18	**0.66**	0.13	-0.09	-0.18	0.14	0.05	0.03	0.20	0.01
27	0.11	0.13	0.08	0.21	**0.57**	0.14	0.07	-0.07	0.08	0.13	0.04	0.14	0.00
36	-0.06	0.12	0.12	0.01	**0.46**	0.20	0.04	0.14	-0.14	-0.07	0.02	-0.17	0.08
69	0.08	0.03	0.06	0.00	**0.71**	0.10	0.13	-0.15	**-0.37**	0.01	0.04	-0.03	0.02
15	0.15	-0.16	0.02	0.19	0.01	0.11	**0.34**	0.13	0.20	**0.36**	0.01	0.00	0.01
29	0.02	0.03	0.23	0.30	0.12	0.03	**0.46**	0.21	0.14	0.01	-0.06	0.20	0.03
30	-0.07	0.07	0.25	0.10	0.03	0.07	**0.28**	0.10	0.13	0.03	0.12	0.13	0.03
31	0.10	0.03	0.03	0.14	0.03	0.10	**0.42**	-0.09	0.00	0.07	0.06	**0.35**	0.11
38	0.04	-0.16	0.17	0.18	-0.27	-0.11	**0.36**	-0.07	0.05	0.02	0.02	0.04	0.13
71	-0.02	0.13	0.00	**-0.38**	0.08	0.24	**0.41**	0.08	0.14	0.14	-0.13	0.11	0.06

	Preoccupied			Autonomous			Dismissing			Unresolved		Structuration	
Item	A	B	C	D	E	F	G	H	I	J	K	L	M
13	0.03	0.06	0.14	0.14	-0.12	0.19	0.04	0.01	**0.44**	0.26	0.06	0.01	0.12
47	0.13	0.07	0.27	0.01	0.04	0.01	0.13	0.16	**0.20**	0.26	0.02	0.14	0.12
50	0.23	0.00	0.04	0.21	0.09	0.23	0.12	0.13	**0.16**	-0.07	0.07	0.13	0.08
57	0.02	0.06	**0.32**	0.23	-0.17	0.19	0.08	0.06	**0.25**	0.10	0.07	0.07	0.16
60	0.08	0.06	0.17	0.04	-0.06	0.19	0.01	-0.04	**0.29**	0.29	-0.09	**0.33**	0.06
67	0.12	0.11	0.09	0.01	0.01	0.18	**0.38**	0.12	**0.31**	0.11	0.00	0.16	0.13
12	-0.13	0.10	0.00	0.08	0.17	0.16	0.03	**0.49**	**0.42**	-0.01	0.01	0.09	-0.01
14	0.03	-0.09	0.10	0.11	-0.26	0.05	0.13	**0.34**	0.25	0.03	-0.07	0.00	0.03
17	-0.09	0.00	0.06	0.03	-0.18	0.17	0.21	**0.37**	0.10	0.15	0.02	0.13	0.13
3	0.03	-0.02	-0.15	0.01	-0.12	0.05	0.06	-0.23	0.16	**0.52**	-0.07	0.11	0.08
33	0.02	0.05	0.12	0.07	0.04	0.07	0.14	0.06	0.12	**0.57**	0.06	-0.09	0.05
45	-0.30	0.09	-0.08	0.10	0.01	0.06	0.04	0.06	0.13	**0.81**	-0.02	0.13	0.12
59	0.01	0.07	0.21	0.16	0.17	0.07	0.20	-0.02	0.00	**0.57**	0.02	-0.05	0.02
61	0.10	0.03	0.13	0.09	0.16	0.29	0.10	-0.02	**0.31**	**0.22**	-0.10	**0.31**	0.07
63	-0.16	0.03	-0.19	0.04	0.15	0.07	0.01	-0.06	0.27	**0.65**	0.04	0.03	0.11
37	0.11	0.14	0.17	0.21	0.01	-0.03	-0.15	0.03	0.06	0.01	**0.94**	0.07	0.20
46	0.05	0.11	0.04	0.05	-0.09	0.23	0.08	-0.01	0.01	0.05	**0.55**	0.05	0.14
51	0.13	0.22	0.07	0.07	0.10	0.12	-0.01	0.04	0.02	-0.16	**0.85**	0.08	0.12
5	-0.05	0.16	0.04	0.11	0.00	0.08	0.11	0.15	0.19	0.06	0.07	**0.56**	0.00
16	-0.12	0.05	0.07	0.23	0.17	0.02	0.15	0.11	0.21	**0.37**	-0.06	**0.52**	0.18
23	-0.07	0.15	-0.15	0.09	0.11	0.10	**0.37**	0.17	0.12	0.22	0.02	**0.72**	-0.04
42	-0.02	0.04	-0.29	0.13	0.06	0.04	0.11	0.08	0.14	0.09	0.00	**0.73**	0.05
44	0.02	0.05	0.12	0.04	0.10	0.05	-0.16	-0.01	-0.07	0.23	0.07	**0.45**	0.01
70	-0.02	0.14	0.10	0.13	0.04	0.07	0.21	0.04	0.10	0.13	0.02	**0.40**	0.08
8	-0.04	0.17	0.07	0.20	0.02	0.17	0.11	-0.03	0.07	0.11	0.02	-0.19	**0.80**
24	-0.26	0.21	0.08	0.06	0.10	0.00	-0.06	0.28	0.03	0.02	0.13	0.21	**0.28**
34	0.01	-0.02	0.15	0.04	0.02	0.18	0.20	-0.03	0.14	0.14	0.09	0.14	**0.82**
43	0.16	0.15	0.07	0.09	0.00	0.17	0.24	0.13	0.09	-0.17	0.10	0.12	**0.30**
49	0.01	0.07	0.06	0.04	0.04	0.14	0.04	-0.12	0.28	0.08	0.12	-0.05	**0.64**
65	-0.05	0.12	0.04	0.06	0.15	0.14	0.02	0.02	0.15	0.05	0.17	0.02	**0.81**

Note. A= Parental intrusion; B= Preoccupation with family; C= Resentment about childhood experiences; D= Parental support; E= Family support; F= Gratitude for support; G= Parental unavailability; H= Family distance; I= Resentment of childhood rejection; J= Parent-related trauma K= Blocked memories; L= Parental abdication; M= Regard for authority.
